# A multilevel Monte Carlo finite element method for the stochastic Cahn–Hilliard–Cook equation

**DOI:** 10.1007/s00466-019-01688-1

**Published:** 2019-02-25

**Authors:** Amirreza Khodadadian, Maryam Parvizi, Mostafa Abbaszadeh, Mehdi Dehghan, Clemens Heitzinger

**Affiliations:** 1 Institute of Applied Mathematics, Leibniz University of Hannover, Welfengarten 1, 30167 Hanover, Germany; 2 Institute for Analysis and Scientific Computing, Vienna University of Technology (TU Wien), Wiedner Hauptstraße 8–10, 1040 Vienna, Austria; 3 Department of Applied Mathematics, Faculty of Mathematics and Computer Sciences, Amirkabir University of Technology, No. 424, Hafez Ave., Tehran, 15914 Iran; 4 School of Mathematical and Statistical Sciences, Arizona State University, Tempe, AZ 85287 USA

**Keywords:** Multilevel Monte Carlo, Finite element, Cahn–Hilliard–Cook equation, Euler–Maruyama method, Time discretization, 35R60, 60H15, 65M60

## Abstract

In this paper, we employ the multilevel Monte Carlo finite element method to solve the stochastic Cahn–Hilliard–Cook equation. The Ciarlet–Raviart mixed finite element method is applied to solve the fourth-order equation. In order to estimate the mild solution, we use finite elements for space discretization and the semi-implicit Euler–Maruyama method in time. For the stochastic scheme, we use the multilevel method to decrease the computational cost (compared to the Monte Carlo method). We implement the method to solve three specific numerical examples (both two- and three dimensional) and study the effect of different noise measures.

## Introduction

The Cahn–Hilliard equation is a robust mathematical model for describing different phase separation phenomena, from co-polymer systems to lipid membranes. The equation is used to model binary metal alloys [1], polymers [2] as well as cell proliferation and adhesion [3]. In material science, when a binary alloy is sufficiently cooled down, we observe a partial nucleation or spinodal decomposition, i.e., the material quickly becomes inhomogeneous. In fact, after a few seconds, material coarsening will be happened [4]. In polymer solutions and blends, the phase separation process is a dynamic process that one phase stable solution separates into two equilibrium phases upon changes in temperature, pressure, concentration, or even flow fields [5]. In these cases, the spinodal decomposition is described by the Cahn–Hilliard model [6].

The equation is a nonlinear partial differential equation of fourth-order in space and first order in time for which an analytical treatment is not possible. There are several numerical techniques to solve the equation including the finite element method (FEM) [7], isogeometric analysis based on finite element method [8], multigrid finite element [9], conservative nonlinear multigrid method [10], least squares spectral element method [11], Monte Carlo methods [12], radial basis functions (RBF) [13] and meshless local collocation methods [14]. A finite element error analysis of the equation is given in [15]. Adaptive finite elements can also be applied to solve the equation using residuals based a posteriori estimates [16, 17].

A difficulty of the numerical analysis of the Cahn–Hilliard equation is the discretization of the fourth-order operator. Here, after converting the fourth-order equation into a system of two second-order equations (by introducing an auxiliary variable) and writing the variational formulation, the Ciarlet–Raviart mixed finite element method is used for the spatial discretization. The method has been implemented for the damped Boussinesq equation by the authors [18] and they considered the convergence rate and the stability for the semi-discretization scheme and the fully discretized method. For the Cahn–Hilliard equation, the technique was used in [19, 20] for the space discretization.

The stochastic Cahn–Hilliard equation was first considered by Cook [21]. The system allows for considering thermal fluctuations directly in terms of the Cahn–Hilliard–Cook (CHC) equation by a conserved noise source term. The thermal fluctuations play an essential role in the early stage of phase dynamics such as initial dynamics of nucleation [22, 23]. Some authors, such as Binder [24] and Pego [25], have expressed the belief that only the stochastic version can correctly describe the whole decomposition process in a binary alloy [26]. In [27], as another numerical approach, the authors employed the direct meshless local Petrov–Galerkin (DMLPG) to solve the stochastic Cahn-Hilliard-Cook and stochastic Swift-Hohenberg equations.

Multilevel Monte Carlo (MLMC) [28] is a simple and efficient computational technique to estimate the expected value of a random process. Using the method enables us to decrease the computational costs noticeably. The multilevel method was implemented to solve the stochastic elliptic equations, e.g., the drift-diffusion-Poisson system with uniformly distributed random variables [29] and quasi-random points [30]. In [31], the convergence and complexity of the MLMC using Galerkin discretizations in space and a Euler–Maruyama discretization in time for the parabolic equations were explained in details. The technique was used in [32] for solving parabolic (heat equation) and hyperbolic (advection equation) driven by additive Wiener noise

Generally, for the time-dependent stochastic problems, the total error consists of the spatial error (due to the finite element method), the time discretization error (due to the Euler–Maruyama technique) and the statistical error (number of samples). We already know that for the space discretization, fine meshes are needed (specifically for the curved surfaces) which lead to the higher computational complexity. The multilevel Monte Carlo method uses hierarchies of meshes for time and space approximations in the sense that the number of samples and mesh sizes (as well as time steps) on the different levels are chosen such that the errors are equilibrated. For the stochastic Cahn–Hilliard–Cook equation, we strive to determine an optimal hierarchy of meshes, number of samples and time intervals which minimize the total computational work. As a result, we give a-priori estimates on the explained error contributions. In this paper, we use the MLMC-FEM for the fourth-order stochastic equations and calculate the mild solution of the Cahn–Hilliard–Cook equation. In fact, we estimate the total computational error according to the three error contributions. Then, we strive to minimize the computational complexity with respect to a given error tolerance. This procedure is compared to the Monte Carlo method.

The rest of the paper is organized as follows. In Sect. 2, we explain the Cahn–Hilliard and the Cahn–Hilliard–Cook equations with their boundary conditions. Then, we describe how the Ciarlet–Raviart mixed finite element can be used to convert the stochastic equation to a system of second-order equations. In Sect. 3, we demonstrate the implementation of the MLMC-FEM for the time-dependent stochastic equations. In Sect. 4, we give three numerical examples according to two different initial conditions. The solutions of the stochastic equation (the concentration) and the optimization (the optimal hierarchies) are given in this section. Finally, the conclusions are drawn in Sect. 5.

## Cahn–Hilliard–Cook equation

J. W. Cahn and J. E. Hilliard proposed the Cahn–Hilliard (CH) equation. The equation is a mathematical physics model that describes the process of phase separation. The CH equation is as follows

1 $$\begin{aligned} \frac{du}{dt} = M \Delta \,( F^{\prime }(u) -\epsilon ^2\Delta u) \qquad \text {in} ~\Omega \times [0,T], \end{aligned}$$

with the Neumann boundary conditions

2 $$\begin{aligned} \frac{\partial u}{\partial \nu }=0,\qquad \frac{\partial \left( -\epsilon ^2\Delta u+F^\prime (u)\right) }{\partial \nu }=0\qquad \text {on}~\partial \Omega \times [0,T]. \end{aligned}$$

We consider the initial condition at $t=0$ as

3 $$\begin{aligned} u(\varvec{x},0)=u_0(\varvec{x}) \qquad \text {for}~ \varvec{x}\in \Omega , \end{aligned}$$

where $\nu$ denotes the unit outward normal of the boundary and $\Omega$ is a bounded domain in $\mathbb {R}^d ~(d=1,2,3)$. The solution *u* is a rescaled density of atoms or concentration of one of the material components where, in the most applications $u\in [-1,1]$. We should note that *M* is the mobility (here a constant) and the variable $\epsilon$ is a positive constant. The equation arises from the Ginzburg–Landau free energy

4 $$\begin{aligned} \mathcal {L}(u)=\int _{\Omega }\left( F(u)+\frac{\epsilon ^2}{2}|\nabla u|^2\right) ~\text {d}\varvec{x}. \end{aligned}$$

The above free energy includes the bulk energy *F*(*u*) and the interfacial energy (the second term). A popular example of a nonlinear function is

5 $$\begin{aligned} F(u)=\frac{1}{4}u^2(1-u)^2. \end{aligned}$$

The Cahn–Hilliard–Cook equation presents a more realistic model including the internal thermal fluctuations. It can be derived from (1) by adding the thermal noise as

6a $$\begin{aligned} \frac{du}{dt}&= M \Delta \,( F^{'}(u) -\epsilon ^2\Delta u)+\sigma \, \xi \qquad&\text {in} ~\Omega \times [0,T], \end{aligned}$$

6b $$\begin{aligned} \frac{\partial u}{\partial \nu }&=0,\quad \frac{\partial \Delta u}{\partial \nu }=0&\text {on} ~\partial \Omega \times [0,T], \end{aligned}$$

where $\xi$ indicates the colored noise (here white noise) and $\sigma$ is the noise intensify measure.

### Ciarlet–Raviart mixed finite element

To construct a mixed finite element approximation of the Cahn–Hilliard–Cook equation, we first find its weak formulation. For this purpose, we define the auxiliary variable

7 $$\begin{aligned} \gamma :=-M\Delta u+F'(u). \end{aligned}$$

Therefore, the Cahn–Hilliard–Cook equation can be rewritten in the form

8a $$\begin{aligned} \gamma&=-M\Delta u+F'(u), \end{aligned}$$

8b $$\begin{aligned} d u&=\nabla \cdot (M\nabla \gamma )+\sigma \, dW, \end{aligned}$$

8c $$\begin{aligned} \frac{\partial u}{\partial \nu }&=\frac{\partial \gamma }{\partial \nu }=0. \end{aligned}$$

The weak formulation of (8) is given by seeking $(u,\gamma )\in H^1_{*}(\Omega )\times H^1_{*}(\Omega )$ such that

9a $$\begin{aligned} (\gamma ,\chi )_\Omega&=\left( M\nabla u,\nabla \chi \right) _{\Omega }\nonumber \\&\quad +\left( F'(u),\chi \right) _{\Omega }\qquad&\forall \,\chi \in H^1_{*}(\Omega ), \end{aligned}$$

9b $$\begin{aligned} \left( d u,\psi \right) _{\Omega }&=-\left( M\nabla \gamma ,\nabla \psi \right) _{\Omega }\nonumber \\&\quad +\sigma \left( d W,\psi \right) _{\Omega }&\forall \,\psi \in H^1_*(\Omega ), \end{aligned}$$

where

10 $$\begin{aligned} H_*^1(\Omega )=\left\{ u\in H^1_*(\Omega )~|\int _{\Omega } u~ \text {d}x=0 \right\} . \end{aligned}$$

Now let $\tau _h$ be a family of triangulations of $\Omega$ into a finite number of elements (simplex) such that

11 $$\begin{aligned} h=\max _{k\in \tau _h} \text {diam}(k). \end{aligned}$$

We assume that each element has at least one face on $\partial \Omega$ and $k_1,k_2\in \tau _h$ have only one common vertex or a whole edge. Now we define

12 $$\begin{aligned} M_h&:=\left\{ v\in C(\Omega )|~v|_k\in \mathbb {P}_n,~n\ge 1~\forall k\tau _h \right\} , \end{aligned}$$

13 $$\begin{aligned} N_h&:=M\cap H_*^1(\Omega ), \end{aligned}$$

and $\mathbb {P}_n$ is the space of all polynomials of degree at most $n\ge 1$. The semi-discrete Galerkin approximation of the solutions (9a)–(9b) may be defined as a pair of approximations $(u_h,\gamma _h)\in N_h \times M_h$ for which the equalities

14a $$\begin{aligned} (\gamma _h,\chi _{_h})_\Omega&=\left( M\nabla u_h,\nabla \chi _h\right) _\Omega \nonumber \\&\quad +\left( F^\prime (u_h),\chi _h\right) _\Omega \quad&\forall \,\chi _{_h}\in M_h, \end{aligned}$$

14b $$\begin{aligned} (du_h,\psi _h)_\Omega&=-\left( M\nabla \gamma _h,\nabla \psi _h\right) _\Omega \nonumber \\&\quad +(dW,\psi _h)_\Omega&\forall \, \psi _h\in N_h, \end{aligned}$$

hold.

### Full discretization scheme

In this section we apply a fully discretize scheme based the mild solution of (8). In order to obtain the fully discretized scheme, we first rewrite the variational formulation of (9) as follows:

Find $(u,\gamma )\in H^1_{*}(\Omega )\times H^1_{*}(\Omega )$ such that

15a $$\begin{aligned}&(\gamma ,\chi )_\Omega \nonumber \\&\quad =(M\nabla u,\nabla \chi )_{\Omega }+(F^\prime (u),\chi )_{\Omega }\qquad \forall ~\chi \in H^1_*(\Omega ), \end{aligned}$$

15b $$\begin{aligned}&(u(t),\psi )_\Omega -(u_0(t),\psi )_\Omega \nonumber \\&\quad =-\int _{0}^{t}(M\nabla \gamma ,\nabla \psi )_\Omega {+}\sigma (W(t),\psi )_\Omega \qquad \forall ~\psi \in H^1_*(\Omega ). \end{aligned}$$

The mixed finite element formulation of (15) is defined by $(u_h(t),\gamma _h(t))\in N_h\times M_h$ such that

16a $$\begin{aligned}&(\gamma _h,\chi _h)_\Omega \nonumber \\&\quad =(M\nabla u_h,\nabla \chi _h)_{\Omega }+(F^\prime (u_h),\chi _h)_{\Omega }\qquad \quad \forall ~\chi _h\in M_h, \end{aligned}$$

16b $$\begin{aligned}&(u_h(t),\psi _h)_\Omega -(u_0(t),\psi _h)_\Omega \nonumber \\&\quad =-\int _{0}^{t}(M\nabla \gamma _h,\nabla \psi _h)_\Omega \nonumber \\&\qquad +\sigma (W(t),\psi _h)_\Omega \quad \forall ~\psi _h \in N_h\quad t\in (0,T]. \end{aligned}$$

Now we can rewrite (8) in the following abstract evolution equation

17 $$\begin{aligned} dX(t)+\left( A^2 X+AF(X)\right) dt&=\sigma d W(t) \qquad t\in (0,T], \end{aligned}$$

18 $$\begin{aligned} X(0)&=X_0, \end{aligned}$$

where *A* is the negative Neumann Laplacian considered as an unbounded operator in the Hilbert space $H=L_2(\Omega )$, which is the generator of an analytic semigroup $(S(t),~t\ge 0)$ on *H* [33]. The initial value $X_0$ is deterministic and *W* is a cylindrical Wiener process in *H* (i.e., the spatial derivative of a space–time white noise) with respect to a filtered probability space $(\Psi ,\mathcal {F},\mathbb {P}, \{F_t\}_{t\ge 0})$ defined as

19 $$\begin{aligned} W(t)=\sum _{j,k=1}^{\infty } \mu _{j,k}^{\frac{1}{2}}\,\beta _{j,k}(t)\,\sin (j\pi x) \sin (k\pi y). \end{aligned}$$

Here, $\left\{ \beta _{j,k}\right\} _{j,k\in \mathbb {N}}$ indicates a family of real-valued, identically distributed independent Brownian motions and $\left\{ \mu _{j,k}\right\} _{j,k\in \mathbb {N}}$ denote the eigenvalues (here, $\mu _{j,k}=1$ since *W*(*t*) is cylindrical) [34]. Therefore, the Cahn–Hilliard–Cook equation has a continuous mild solution

20 $$\begin{aligned} X(t)&=S(t)X_0+\int _{0}^{t}A S(t-s)F(X(s))~\text {d}s\nonumber \\&\quad +\sigma \int _{0}^{t} S(t-s)~\text {d}W(s), \end{aligned}$$

where $t\in [0,T]$, $X:[0,T]\times \Omega \rightarrow H$ and $S(t)=\text {e}^{-tA^2}$ used as the analytic semigroup generated by $-A^2$. The existence of the mild solution *X* was shown in [35]. Considering $\Vert X_0\Vert _{L^2(\Omega ,H)}\le +\infty$, for all $t\in [0,T]$ the solution *X* satisfies [31]

21 $$\begin{aligned} \Vert X(t)\Vert _{L^2(\Omega ,H)}\le C(T) \left( 1+\Vert X_0\Vert _{L^2(\Omega ,H)}\right) , \end{aligned}$$

where *C* is a constant which depends on *T*. Also, for $0\le s<t\le T$, there exists a constant *C*(*T*) such that the mild solution satisfies the inequality [31]

22 $$\begin{aligned} \Vert X(t)-X(s)\Vert _{L^2(\Omega ,H)}\le C(T)\sqrt{t-s}\left( 1+\Vert X_0\Vert _{L^2(\Omega ,H)}\right) . \end{aligned}$$

In order to estimate the mild solution we use finite elements for space discretization and the semi-implicit Euler–Maruyama scheme in time direction. Let us assume that $V_\ell$ ($\ell \in \mathbb {N}_0$) is a nested family of finite element subsequences of *H* with refinement level $\ell >0$ and refinement size $h_\ell ~(\ell \in \mathbb {N}_0$). Defining the analytic semigroup $S_{\ell }=\text {e}^{-tA_\ell ^2}$, for $t\in T$, the semidiscrete problem (20) has the form

23 $$\begin{aligned} X_\ell (t)&=\text {e}^{-tA_\ell ^2}X_\ell (0)+\int _{0}^{t}A_\ell \text {e}^{-(t-s)A_\ell ^2} F(X_\ell (s))~\text {d}s\nonumber \\&\quad +\sigma \int _{0}^{t}\text {e}^{-(t-s)A_\ell ^2} ~\text {d}W(s). \end{aligned}$$

For the time direction, we approximate the time discretization with step sizes $\delta t^\zeta =Tr^{-\zeta }$ where $r>1$. Therefore, for $\zeta \in \mathbb {N}_0$, we define the sequence

24 $$\begin{aligned} \Theta ^\zeta :=\left\{ t_k^\zeta =Tr^{-\zeta }k=\delta t^\zeta k,~k=0,\ldots ,r^\zeta \right\} \end{aligned}$$

of equidistant time discretization. In the computational geometry $(\Omega )$, we estimate the mild solution *X*, with a finite element discretization. In other words, we suppose that the domain can be partitioned into quasi-uniform triangles or tetrahedra such that sequences $\{\tau _{h_{\ell }} \}_{{\ell }=0}^{\infty }$ of regular meshes are obtained. For any $\ell \ge 0$, we denote the mesh size of $\tau _{h_{\ell }}$ by

$$\begin{aligned} h_{\ell } := \max _{K\in \tau _{h_\ell }} {\text {diam}} K. \end{aligned}$$

Uniform refinement of the mesh can be achieved by regular subdivision. This results in the mesh sizes

25 $$\begin{aligned} h_\ell := r^{-\ell } h_0, \end{aligned}$$

where $h_0$ denotes the mesh size of the coarsest triangulation and $r>1$ is independent of $\ell$.

## Multilevel Monte Carlo finite element method

The Monte Carlo method is a simple and efficient computational technique to solve SPDEs. As already mentioned, we use Euler–Maruyama to solve the equation on [0, *T*] and the finite element method for the space discretization. In order to obtain the mean square error (MSE) of $\varepsilon$, we require $\delta t=\mathcal {O}(\varepsilon )$ (for the time discretization). The Monte Carlo error (statistical error) is $\mathcal {O}(1/\sqrt{M})$ (where *M* is the number of samples) which yields $M^{-1}=\mathcal {O}(\varepsilon ^{2})$. Using a finite element scheme also gives rise to $\mathcal {O}(\varepsilon ^{-d/\alpha })$, where $\alpha$ is the convergence rate of the discretization error. Therefore, by taking *M* samples, $T/\delta t$ time steps and *h* as the mesh size, we have the following total cost

26 $$\begin{aligned} \mathcal {W}=\mathcal {O}\left( \varepsilon ^{-(2+1+d/\alpha )}\right) . \end{aligned}$$

It is obvious that for high dimensional geometries (i.e., $d=2,3$), the computational cost increases noticeably.

Multilevel Monte Carlo finite element method (MLMC-FEM) is an efficient alternative to the Monte Carlo method to decrease the cost. In the time discretization, the general idea of the technique is using a hierarchy of the time steps, i.e., $\delta t^\ell$ ($\ell \in \mathbb {N}_0$) at different levels (instead of a fixed time step). For the space discretization, we use the mesh refinement (25) to obtain the mesh size at level $\ell$ which leads to

27 $$\begin{aligned} h_0>h_1>\cdots>h_{L-1}>h_{L}. \end{aligned}$$

In this section we strive to estimate the expectation of the mild solution on level *L*. First, for a given Hilbert space $(H, \Vert \cdot \Vert _H)$ the space, $L^2(\Omega ;H)$ is defined to be the space of all measurable functions $\mathcal {Y}:\Psi \rightarrow H$ such that

28 $$\begin{aligned} \Vert \mathcal {Y} \Vert _{L^p(\Omega ;H)} = \mathbb {E}\Big [\Vert \mathcal {Y} \Vert _H^2 \Big ]^{1/2}. \end{aligned}$$

For $\mathcal {Y}\in L^2(\Omega ;H)$ the standard Monte Carlo estimator $E_M[\mathcal {Y}]$ can be defined as

29 $$\begin{aligned} E_{M}[\mathcal {Y}]: = \frac{1}{M} \sum _{i=1}^{M} \hat{\mathcal {Y}}^{(i)}, \end{aligned}$$

where for $i=1,\ldots ,M$, $\mathcal {Y}^{(i)}$ indicated a sequence of i.i.d. copies of $\mathcal {Y}$. Let $\mathcal {Y}_\ell ~(\ell \in \mathbb {N}_0)$ be a sequence of random variables such that $\mathcal {Y}_\ell \in V_\ell$, we can write $\mathcal {Y}_L$ as

30 $$\begin{aligned} \mathcal {Y}_L=\mathcal {Y}_0+\sum _{\ell =1}^{L}\left( \mathcal {Y}_\ell -\mathcal {Y}_{{\ell }-1}\right) , \end{aligned}$$

taking expected value of the above equality leads to

31 $$\begin{aligned} {\mathbb {E}}[\mathcal {Y}_L]= & {} {\mathbb {E}}[\mathcal {Y}_0]+{\mathbb {E}}\left[ \sum _{{\ell }=1}^{L} (\mathcal {Y}_{{\ell }}-\mathcal {Y}_{{{\ell }-1}})\right] \nonumber \\= & {} {\mathbb {E}}[\mathcal {Y}_{0}]+\sum _{{\ell }=1}^{L}{\mathbb {E}} [\mathcal {Y}_{{\ell }}-\mathcal {Y}_{{{\ell }-1}}]. \end{aligned}$$

In order to approximate ${\mathbb {E}}[\mathcal {Y}_{{\ell }}-\mathcal {Y}_{{{\ell }-1}}]$ we can use the Monte Carlo estimator $E_{M_\ell }[\mathcal {Y}_{{\ell }}-\mathcal {Y}_{{{\ell }-1}}]$ (i.e., expectation of the difference of $\mathcal {Y}_\ell$ and $\mathcal {Y}_{\ell -1}$) with independent number of samples $M_\ell$ at level $\ell$. Therefore, (31) can be estimated as

32 $$\begin{aligned} E^{L}[\mathcal {Y}_L]=E_{M_0}[\mathcal {Y}_0]+E_{M_\ell } \left[ \sum _{{\ell }=1}^{L}(\mathcal {Y}_{{\ell }}-\mathcal {Y}_{{{\ell }-1}})\right] , \end{aligned}$$

In this part, we first provide the error bound for the single level Monte Carlo finite element. Then, using the obtained results, we achieve the error bound of the multilevel Monte Carlo considering the principal discretization error, i.e., spatial discretization (using finite element method), time stepping errors (due to the Euler–Maruyama technique) and statistical (sampling) error.

### Lemma 1

[29] For any number of samples $M \in \mathbb {N}$ and for $\mathcal {Y} \in L^2(\Omega ;H)$, the inequality

33 $$\begin{aligned} \Vert {\mathbb {E}}[\mathcal {Y}]-E_M[\mathcal {Y}] \Vert _{L^2(\Omega ;H)} {=} M^{-1/2} \sigma [\mathcal {Y}]{\le } M^{-1/2} \Vert \mathcal {Y}\Vert _{L^2(\Omega ;H)} \end{aligned}$$

holds for the MC error, where $\sigma [\mathcal {Y}] := \Vert \,\mathbb {E}[\mathcal {Y}]-\mathcal {Y}\,\Vert _{L^2(\Omega ;H)}$.

According to Lemma 1 for $\ell ,~\zeta \in \mathbb {N}_0$ and $t\in \Theta ^\zeta$, we have the inequality

34 $$\begin{aligned}&\Big \Vert \mathbb {E}\left[ X_{\ell ,\zeta }(t)\right] -E_M\left[ X_{\ell ,\zeta }(t)\right] \Big \Vert _{L^2(\Omega ;H)}\nonumber \\&\quad \le \frac{1}{\sqrt{M}}\Vert X_{\ell ,\zeta }(t)\Vert _{L^2(\Omega ;H)}, \end{aligned}$$

where $X_{\ell ,\zeta } (t)$ is the discrete mild solution at level $\ell$ and time interval $\zeta$. In order to estimate the discretization error which stems from the spatial discretization and time stepping we define the following lemma.

### Lemma 2

[31] Let *X* be the solution of (20) and $X_{\ell ,\zeta }$ be the sequence of discrete mild solution (i.e., the solution of (23)). Then, there is a constant *C*(*T*) such that for all $\ell ,\zeta \in \mathbb {N}_0$, we have

35 $$\begin{aligned}&\sup _{t\in \Theta ^\zeta }\Vert X(t)-X_{\ell ,\zeta }(t)\Vert _{L^2(\Omega ;H)}\nonumber \\&\quad \le C(T)\left( h_\ell +\sqrt{\delta t ^\zeta } \right) \left( 1+\Vert X_0 \Vert _{L^2(\Omega ;H)}\right) . \end{aligned}$$

Hence, the total computational error is given by [31]

36 $$\begin{aligned}&\sup _{t\in \Theta ^\zeta }\left\| \,\mathbb {E}\left[ X(t)\right] -E_M\left[ X_{\ell ,\zeta }(t)\right] \,\right\| _{L^2(\Omega ;H)}\nonumber \\&\quad \le C(T)\left( h_\ell +\sqrt{\delta t ^\zeta }+\frac{1}{\sqrt{M}}\, \right) \left( 1+\Vert X_0 \Vert _{L^2(\Omega ;H)}\right) . \end{aligned}$$

In order to prove, we add and subtract the term $\mathbb {E}[X_{\ell ,\zeta }(t)]$ to the left side and use the triangle inequality, Lemma 1, Lemma 2 and (34) to obtain the error bound. Now we couple the space and time errors and choose $\delta t^\zeta \simeq h_\ell ^2$ (i.e., $\delta t^\zeta =\mathcal {O}(h_\ell ^2)$ and $\mathcal {O}(\delta t^\zeta )=h_\ell ^2$). Therefore, the total work $\mathcal {W}_\ell$ for the given spatial discretization level $\ell \in \mathbb {N}_0$ is estimated by

37 $$\begin{aligned} \mathcal {W}_\ell \simeq h_\ell ^{-d}h_\ell ^{-2} M. \end{aligned}$$

After estimating the error bounds for single level Monte Carlo, we provide the multilevel Monte Carlo error bounds. By using $\zeta =2\ell$ (due to $\delta t^\zeta \simeq h_\ell ^2$), for $\ell \in \mathbb {N}_0$ we consider $h_\ell \simeq r^{-\ell }$ and define $\delta t^\ell := T r^{-2\ell }$. Therefore, for the full discretization in the multilevel setting, we redefine (24) as

38 $$\begin{aligned} \Theta ^\ell :=\left\{ t_{k(\ell )}^\ell =Tr^{-2\ell }k(\ell )=\delta t^\ell k(\ell ),~k(\ell )=0,\ldots ,r^{2\ell } \right\} , \end{aligned}$$

and we define the multilevel Monte Carlo estimator

39 $$\begin{aligned}&E^L[X(t_{k(L)}^L)]:=E_{M_0}[X_0(t_{k(L)}^L)]\nonumber \\&\quad +\sum _{\ell =1}^{L} E_{M_\ell }[X_\ell (t_{k(L)}^L)-X_{\ell -1}(t_{k(L)}^L)]. \end{aligned}$$

The computational error is given by

40 $$\begin{aligned} \mathcal {E}=\sup _{t\in \Theta ^L}\left\| \,\mathbb {E}[X(t)]-E^L[X_L(t)]\, \right\| _{L^2(\Omega ;H)}. \end{aligned}$$

For fixed $L\in \mathbb {N}$ and any chosen $t_{k(L)}^L \in \Theta ^L$, we split the error into two parts, i.e., discretization error and statistical error (see Theorem 4.5 in [31]), to obtain

41 $$\begin{aligned}&\Vert \mathbb {E}[X(t_{k(L)}^L)]-E^L[X_L(t_{k(L)}^L)]\Vert _{L^2(\Omega ;H)}\nonumber \\&\quad \le \Vert X(t_{k(L)}^L)-X_L(t_{k(L)}^L)\Vert _{L^2(\Omega ;H)}\nonumber \\&\qquad +\frac{1}{\sqrt{M_0}}\Vert X_0(t_{k(L)}^L)\Vert _{L^2(\Omega ;H)}\nonumber \\&\qquad +\sum _{\ell =1}^{L} \frac{1}{\sqrt{M_\ell }}\Vert X_\ell (t_{k(L)}^L)-X_{\ell -1}(t_{k(L)}^L)\Vert _{L^2(\Omega ;H)} \end{aligned}$$

Now we make the following convergence assumptions for the prescribed errors. The below assumption is used to estimate the convergence rate of the discretization error

**Fig. 1:**
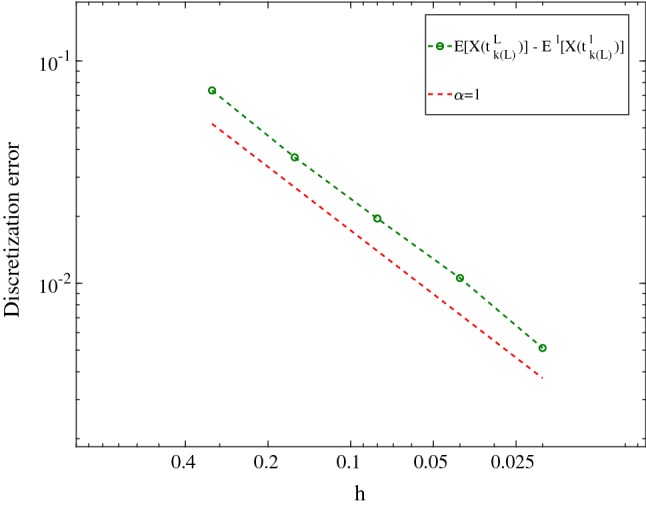
The discretization error as a function of different mesh sizes where $\alpha =0.95$ as the convergence rate is obtained

**Table 1:** The optimal hierarchies of MLMC-FEM with respect to different prescribed errors

$\varepsilon$	$h_0$	*r*	$M_0$	$M_1$	$M_2$	$M_3$	$M_4$
0.100	0.764	1.458	73	50	15	–	–
0.050	0.615	1.568	280	138	31	–	–
0.020	0.461	1.726	1672	524	85	–	–
0.010	0.370	1.856	6474	1445	184	–	–
0.005	0.580	1.990	187,700	46,448	4615	459	–

**Table 2:** The optimal values of MC-FEM with respect to different prescribed errors

$\varepsilon$	0.1	0.05	0.02	0.01	0.005
*h*	0.108	0.052	0.020	0.010	0.005
*M*	3	12	73	289	1 152

**Fig. 2:**
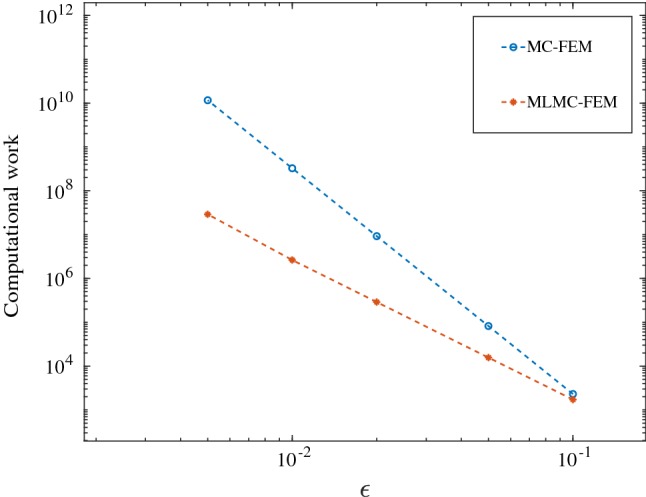
A comparison between the optimal work of MLMC-FEM and MC-FEM showing the efficiency of the multilevel technique is pronounced

42 $$\begin{aligned} \Vert X(t_{k(L)}^L)-X_L(t_{k(L)}^L)\Vert _{L^2(\Omega ;H)}&\le C_1 h_L^\alpha . \end{aligned}$$

Regarding the statistical error, the next assumptions

43 $$\begin{aligned} \Vert X_0(t_{k(L)}^L)\Vert _{L^2(\Omega ;H)}&\le C_2, \end{aligned}$$

44 $$\begin{aligned} \Vert X_\ell (t_{k(L)}^L)-X_{\ell -1}(t_{k(L)}^L)\Vert _{L^2(\Omega ;H)}&\le C_3 h_{\ell } \end{aligned}$$

are made. Hence, the total error can be estimated as

45 $$\begin{aligned} \mathcal {E}\le C_1 h_L^\alpha +\frac{C_2}{\sqrt{M_0}}+C_3\sum _{\ell =1}^{L}\frac{ h_{\ell }}{\sqrt{M_\ell }}. \end{aligned}$$

The total work can be estimated by summing up the work of each level, i.e.,

**Fig. 3:**
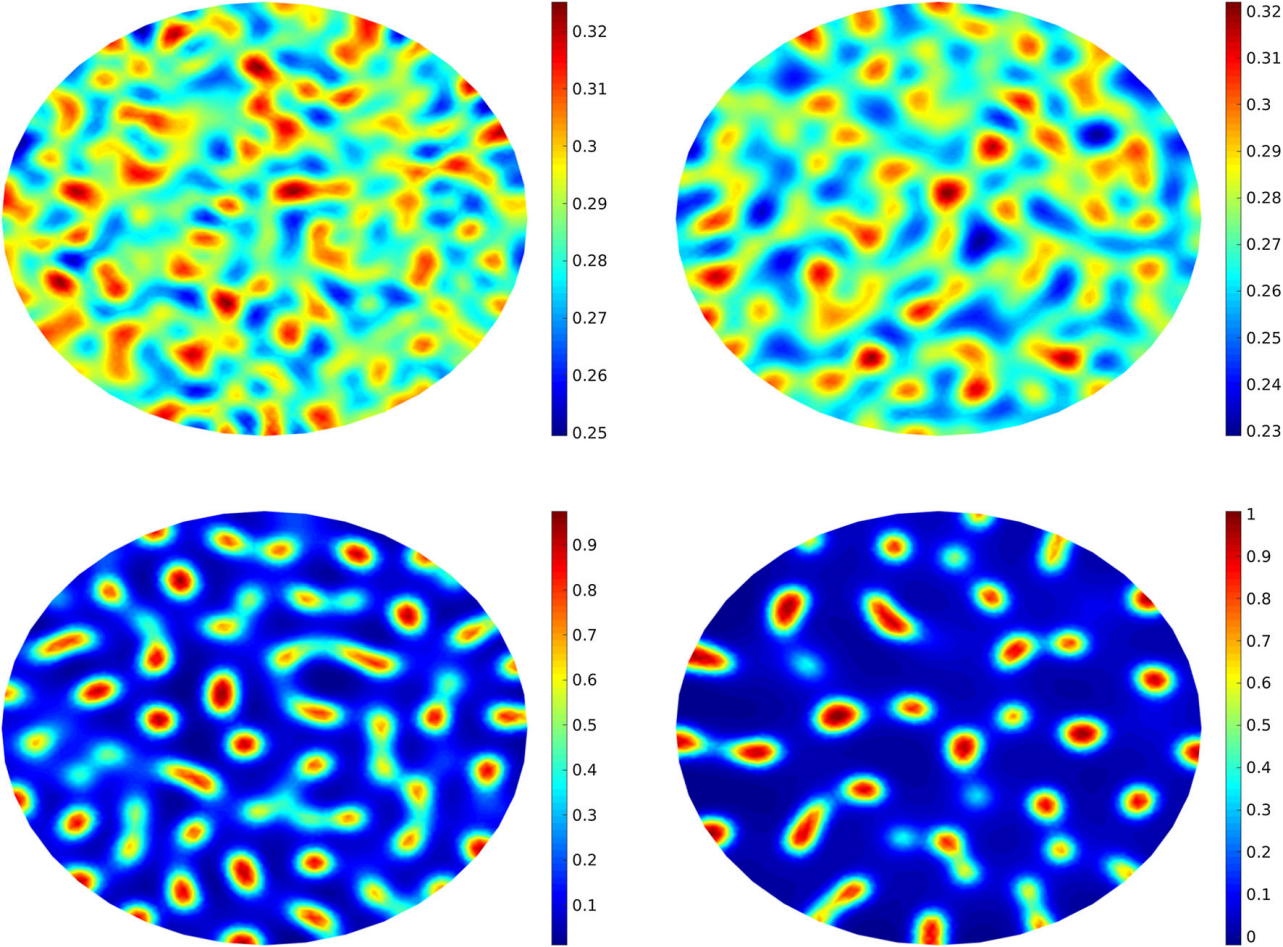
The solution of the stochastic CHC equation at $T=0.1$ (top left), $T=0.5$ (top right), $T=3$ (bottom left) and $T=15$ (bottom right)

**Fig. 4:**
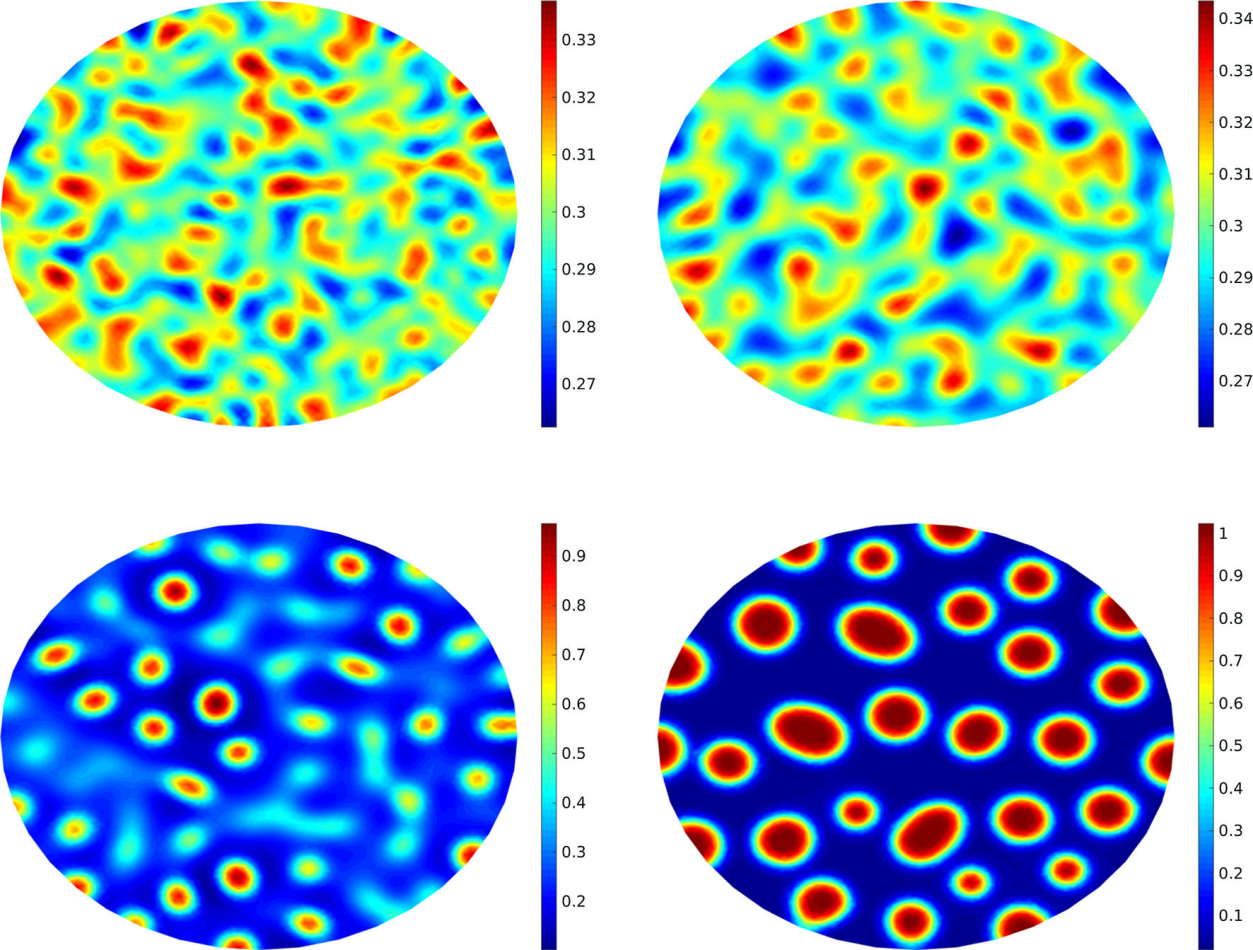
The solution of the Cahn–Hilliard equation ($\sigma =0$) at $T=0.1$ (top left), $T=0.5$ (top right), $T=3$ (bottom left) and $T=15$ (bottom right)

**Fig. 5:**
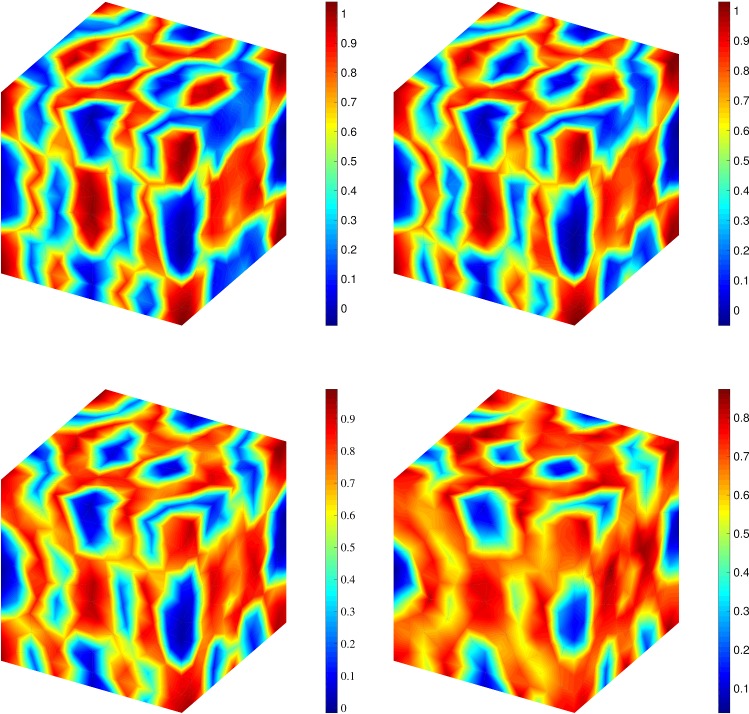
A comparison between different noise intensify measure, i.e., $\sigma =0$ (top left), $\sigma =0.1$ (top right), $\sigma =0.2$ (bottom left) and $\sigma =0.3$ (bottom right) at $T=1$

46 $$\begin{aligned} \mathcal {W}=\sum _{\ell =0}^{L}\mathcal {W}_\ell =\sum _{\ell =0}^{L}\mu _\ell h_\ell ^{-d}h_\ell ^{-2} M_\ell . \end{aligned}$$

Now we define an optimization problem which minimizes the computational work (46) such that the error is less or equal than a given error tolerance $(\varepsilon )$. In other words, for estimating the mild solution on level *L*, we estimate the optimal hierarchies of $\left( h_\ell ,M_\ell ,L\right) _{\ell =0}^{\ell =L}$ such that

47 $$\begin{aligned} \begin{aligned}&\underset{M_\ell ,h_0,r}{\text {minimize}}&f(M_\ell , h_0,r,L) := \sum _{\ell =0}^{L} \mathcal {W}_\ell \\&\text {subject to}&g(M_\ell ,h_0,r,L):= C_1 h_L^\alpha +\frac{C_2}{\sqrt{M_0}}\\&\qquad +C_3\sum _{\ell =1}^{L}\frac{ h_{\ell }}{\sqrt{M_\ell }} \le \varepsilon . \end{aligned} \end{aligned}$$

In the problem we have $M_\ell>1, h_0>0$ and $r>1$. The exponent ($\alpha$) as well as the coefficients ($C_1,~ C_2,~C_3$) must be determined analogously. Finally, we should note that for Monte Carlo method the optimization problem with respect to (26) can be written as

48 $$\begin{aligned} \begin{aligned}&\underset{M,h}{\text {minimize}}&f(M,h):= M h^{-(d+1)}\\&\text {subject to}&g(M,h):= C_1 h^{\alpha }+\frac{C_2}{\sqrt{M}} \le \varepsilon , \end{aligned} \end{aligned}$$

where again the optimization problem is over $M>1$ and $h>0$.

**Fig. 6:**
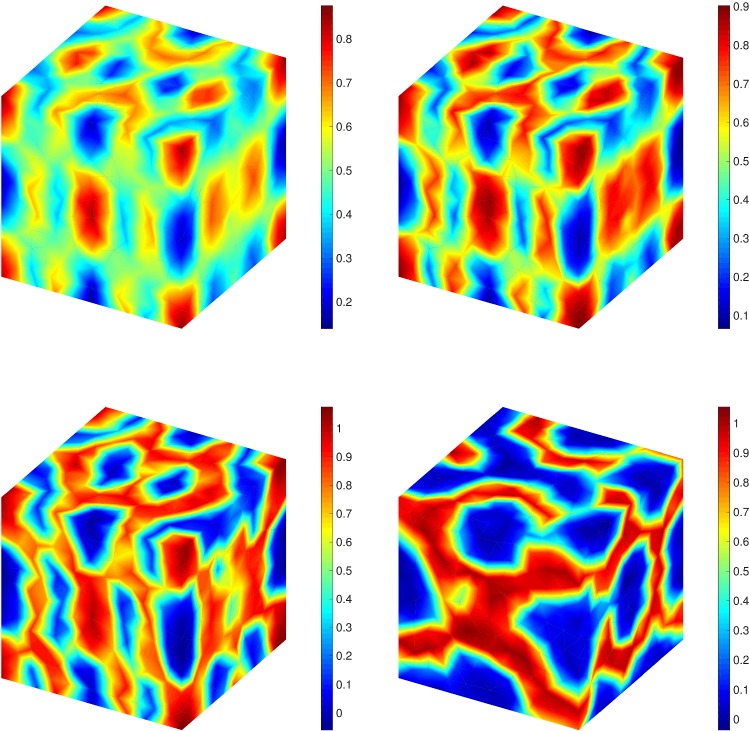
The expected value of the solution of CHC equation at $T=0.1$ (top left), $T=0.4$ (top right), $T=2$ (bottom left) and $T=10$ (bottom right). Here, $h=0.2$ and $\sigma =0.1$

**Fig. 7:**
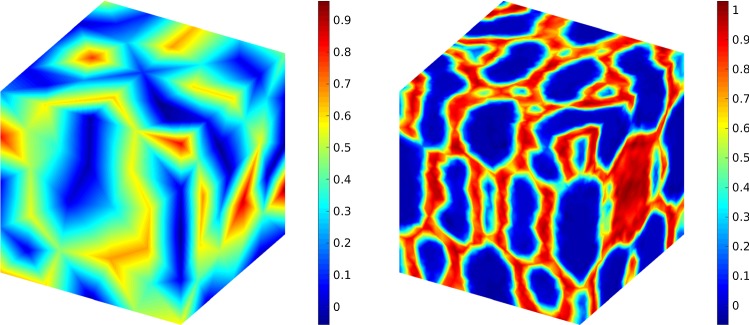
A comparison between two mesh sizes $h=0.5$ (left) and $h=0.1$ (right) at $T=5$ for the stochastic Cahn–Hilliard–Cook equation

**Fig. 8:**
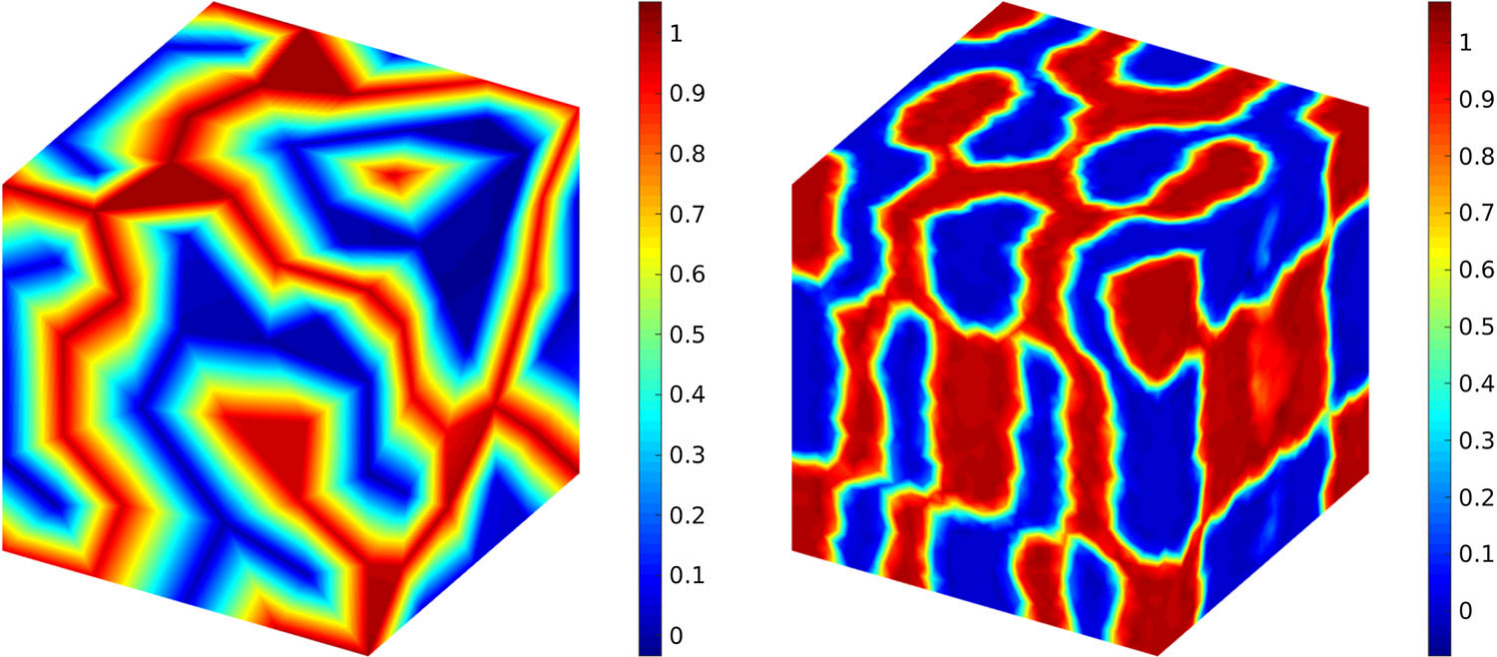
A comparison between two mesh sizes, i.e., $h=0.5$ (left) and $h=0.1$ (right) at $T=5$ for the deterministic Cahn–Hilliard equation

**Fig. 9:**
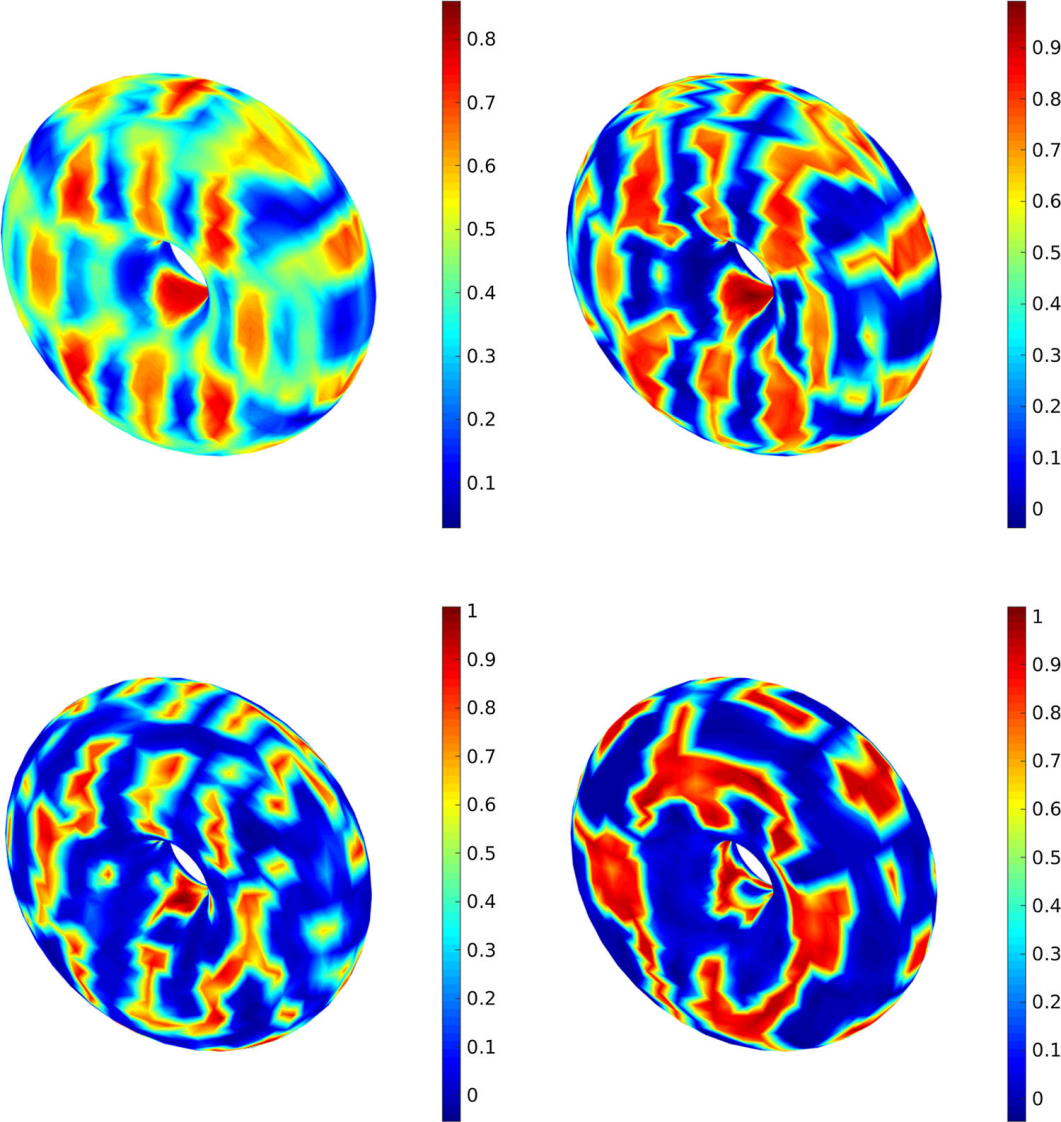
The evolution of the solution of the times $T=0.1$ (top left), $T=0.5$ (top right), $T=3$ (bottom left) and $T=20$ (bottom right)

## Numerical results

In this section, we present three numerical examples of the stochastic Cahn–Hilliard–Cook equations where in all cases the optimal MLMC-FEM is used to obtain the solution. Due to the fact that the examples are real-world problems, their exact solutions are not given. The simulations are performed using MATLAB 2017b software on an Intel Core i7 machine with $32\,\mathrm {GB}$ of memory. In all examples, $\epsilon =0.01$ is used and the constant mobility $M=0.25$ is applied. For the nonlinear term (i.e., $F'(u)$), we use Newton’s method where several iterations are needed to reach the stopping tolerance (here $TOL=1\times 10^{-8}$). In each iteration, the built-in direct solver is employed to solve the linearized system.

### A 2D example

As the first example, we take $u_0=0.25+0.1{\omega }$ as the initial condition. The random variable ${\omega }$ is uniformly distributed between 0 and 1. The computational geometry ($\Omega$) is a circle with radius $r=2$ and zero center point. As the first step, we try to solve the optimization problems (for MLMC-FEM and MC-FEM). This will enable us to find the optimal number of samples and mesh sizes. As explained in Sect. 2.1, we use the Ciarlet–Raviart mixed finite element with P1 elements. The estimation of the exponent $\alpha$ is crucial, however, it relates to the order of polynomials. Due to the fact that the exact solution of the stochastic equation is not available, we calculate the error between different mesh sizes and $h=0.01$ (as the reference solution) at $T=5$. Figure 1 depicts $\alpha$ with respect to different mesh sizes (here uniform refinement). The simulations show $\alpha =0.952$, $C_1=0.51$, where the exponent agrees very well with the order of P1 finite element technique (linear elements). The rest of the coefficients is estimated as $C_2=0.066$, $C_3=0.223$. Now we are ready to solve the optimization problem (47) with respect to the aforementioned parameters. In order to solve the optimization problem, we apply interior method where the details of the technique are given in [29]. The optimal hierarchies of the MLMC-FEM are shown in Table 1.

As the next step, in order to compare the efficiency of the multilevel setting with the Monte Carlo simulation, we solve the optimization given in (48). Again, the optimal mesh size and the optimal number of samples are given in Table 2 where the same convergence rate ($\alpha$) is used. Finally, we draw a comparison between MLMC-FEM and MC-FEM which is shown in Fig. 2. The results indicate that the multilevel method costs approximately $\mathcal {O}(\varepsilon ^{-3.27})$ and the computational work of Monte Carlo sampling is $\mathcal {O}(\varepsilon ^{-5.1})$. The comparison indicates noticeably the efficiency of the MLMC-FEM.

Finally, we compare the evolution of the concentration $\mathbb {E}[u(T)]$ at different times (from $T=0.2$ to $T=15$) (with $\sigma =0.1$) where the obtained results are depicted in Fig. 3. It is shown that from $T=0.2$ to $T=1$, a slow coarsening happens. Here, we use $\varepsilon =0.05$ in the sense that the solution at the last level (here $L=2$) is shown in the figure (see Table 1 for the optimal mesh size and number of samples). In order to study the noise effect we solve the deterministic equation with the same mesh size as illustrated in Fig. 4.

### The 3D examples

Here we choose a more complicated example and use MLMC-FEM and CR-MFE to obtain the solution (expected value) of CHC equation in a cubic geometry, i.e., $\Omega =[0,2]\times [0,2]\times [0,2]$. The initial condition is

49 $$\begin{aligned} u_0(x,y,z)&:=0.5 + 0.17 \cos (\pi x )\cos (2\pi y )\cos (\pi z) \nonumber \\&\;\quad + 0.2 \cos (3\pi x )\cos (\pi y )\cos (\pi z), \end{aligned}$$

where (*x*, *y*, *z*) is a point on the cube. The same procedure for solving the optimization problem can be used, however, due to the three-dimensional structure we set $d=3$. We should note that as $\zeta =2\ell$, we define the optimal time interval as $\delta t^\zeta \simeq h_\ell ^2$. First, we consider the effect of the noise intensify measure in the sense that the deterministic case ($\sigma =0$) is compared with the stochastic equation ($\sigma =0.1,~0.2,~0.3$). The results are shown in Fig. 5 at $T=1$. Clearly, higher $\sigma$ affects the concentration mostly. Similar to the 2D example, we consider the effect of time on the concentration. The results are shown in Fig. 6 for different times from $T=0.1$ to $T=10$. It illustrates that the initial homogeneous phase quickly segregates (at $T=0.1$), however, after the segregation the domain starts to slowly coarsen in time.

In the next step, we use the Monte Carlo finite element method to compare the effect of the number of grids. Here, two mesh sizes, i.e., $h=0.5$ (with $137^3$ nodes) and $h=0.1$ (with $6651^3$ nodes) are employed and the results are shown in Figs. 7 and 8 for stochastic and deterministic cases, respectively. We solved the CHC equation with $\sigma =0.15$ and compared its solution with the deterministic case ($\sigma =0$) at time $T=5$. It shows that the mesh size does not considerably affect the solution.

**Fig. 10:**
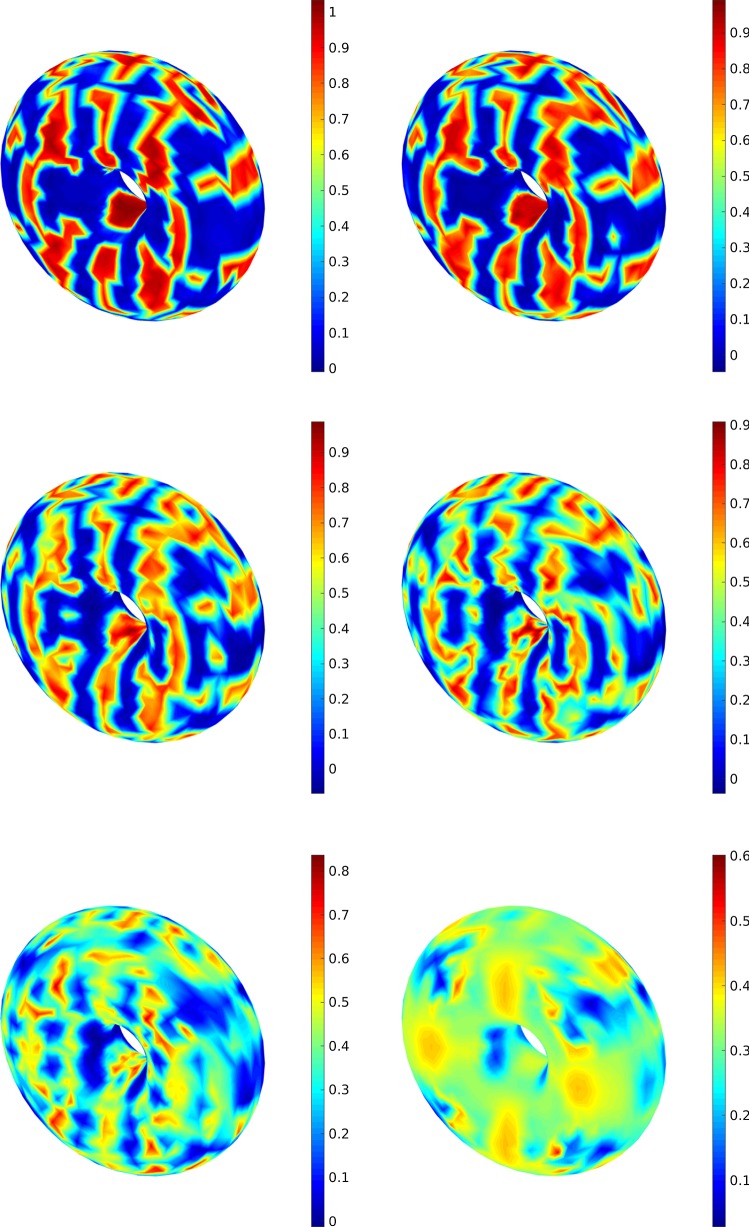
A comparison between different noise intensify measure, i.e., $\sigma =0$ (top left), $\sigma =0.1$ (top right), $\sigma =0.2$ (middle left) and $\sigma =0.3$ (middle right), $\sigma =0.4$ (bottom left) and $\sigma =0.5$ (bottom right) at $T=1$

Finally, we consider the second 3D example (a torus). The first comparison is regarding the evolution of the concentration which is illustrated in Fig. 9 where in the simulations $\sigma =0.1$ is used. In the second case, we study the effect of different noise measures from deterministic case to stochastic case with $\sigma =0.5$ at $T=1$. Here, the results are shown in Fig. 10. The simulations point out that the effect of $\sigma =0.4$ and $\sigma =0.5$ on the concentrations are more tangible.

## Conclusions

In this paper, we considered the Cahn–Hilliard and Cahn–Hilliard–Cook equations as forth-order time-dependent equations. As the first step, after defining an auxiliary variable, we converted the equation into a system of second-order time-dependent problems. Then, we presented a variational formulation for the system and used the Ciarlet–Raviart mixed finite element method. Afterwards, we rewrote the equation as a stochastic ODE in order to estimate its mild solution *u*(*t*).

We have already shown that for the stochastic time-dependent problems, the computational cost of the Monte Carlo finite element method is $\mathcal {O}(\varepsilon ^{-(2+1+d/\alpha )})$. Applying the multilevel technique for this problem reduces noticeably the computational costs. In a two-dimensional problem, the optimal hierarchies $\left( h_\ell ,r_\ell ,M_\ell ,\delta t^\ell \right) _{\ell =0}^{\ell =L}$ reduce the complexity to $\mathcal {O}(\varepsilon ^{-3.27})$ as certified in numerical example.

We showed three numerical examples with two specific initial conditions. We estimated the solution of stochastic/deterministic Cahn–Hilliard equation for different time intervals. As a result, we demonstrated distinctive coarsening and phase separation. For the stochastic equation, we studied the effect of noise measure, for showing that more $\sigma$ intensifies the noisy concentration.
